# Dapsone-Induced Methemoglobinemia Presenting Concomitantly With COVID-19 Pneumonia and Pulmonary Embolism: A Case Report

**DOI:** 10.7759/cureus.51830

**Published:** 2024-01-08

**Authors:** Leena Alhusari, Marlena Pigliacampi, Yara Alshawabkeh, Teseir Hamdani, Taysir Bsiso, Bisher Mustafa, Larry Dial

**Affiliations:** 1 Internal Medicine, Marshall University Joan C. Edwards School of Medicine, Huntington, USA

**Keywords:** hemoglobin disorders, dapsone toxicity, acute hypoxic respiratory failure, dapsone induced methemoglobinemia, dapsone side effect

## Abstract

Acquired methemoglobinemia is a treatable condition that is often clinically subtle and can be missed on routine clinical assessment. We present a 73-year-old male who was evaluated in the emergency department with worsening respiratory symptoms requiring oxygen. He tested COVID-19 positive and had new pulmonary emboli evident on his CT chest. The patient was on dapsone therapy as a treatment for bullous pemphigoid. The discrepancy between his oxygen levels on the pulse oximeter and blood gas was noted and was treated with 3% methylene blue for dapsone-induced methemoglobinemia. The patient received treatment for COVID-19 pneumonia and pulmonary emboli. Our case demonstrates that dapsone-induced methemoglobinemia can present concomitantly with other more common causes of acute hypoxic respiratory failure. It is noteworthy for physicians to maintain a high index of suspicion for oxygen level discrepancy in hypoxic patients and consider the possibility of acquired methemoglobinemia. Hence, earlier detection and treatment of the etiology of tissue hypoxia.

## Introduction

Methemoglobinemia is diagnosed when blood’s methemoglobin concentration is greater than the normal 1%-3% [1,2]. The iron in hemoglobin is oxidized to its ferric state (Fe 3+) causing red blood cells to have an increased affinity for oxygen, shifting the oxygen dissociation curve left. Thus, less oxygen gets released causing functional anemia and eventually tissue hypoxia [3]. We present a case study that analyzes the course of disease and treatment for an elderly patient with dapsone-induced methemoglobinemia concomitant with COVID-19 acute hypoxic respiratory failure. We encourage physicians and healthcare workers to maintain a high index of suspicion for oxygen level discrepancy in hypoxic patients and consider the possibility of acquired methemoglobinemia. Hence, proper management.

## Case presentation

We present a case of a 73-year-old male who was evaluated in the Emergency Department (ED) for having shortness of breath of two weeks onset with worsening respiratory status and increased breathing effort for which he presented to ED for evaluation. He was recently diagnosed with bullous pemphigoid three months ago and was started on dapsone 25 mg/day. He also complained of sore throat and nasal drainage one day prior to ED presentation and tested COVID-19 positive.

**Figure 1:**
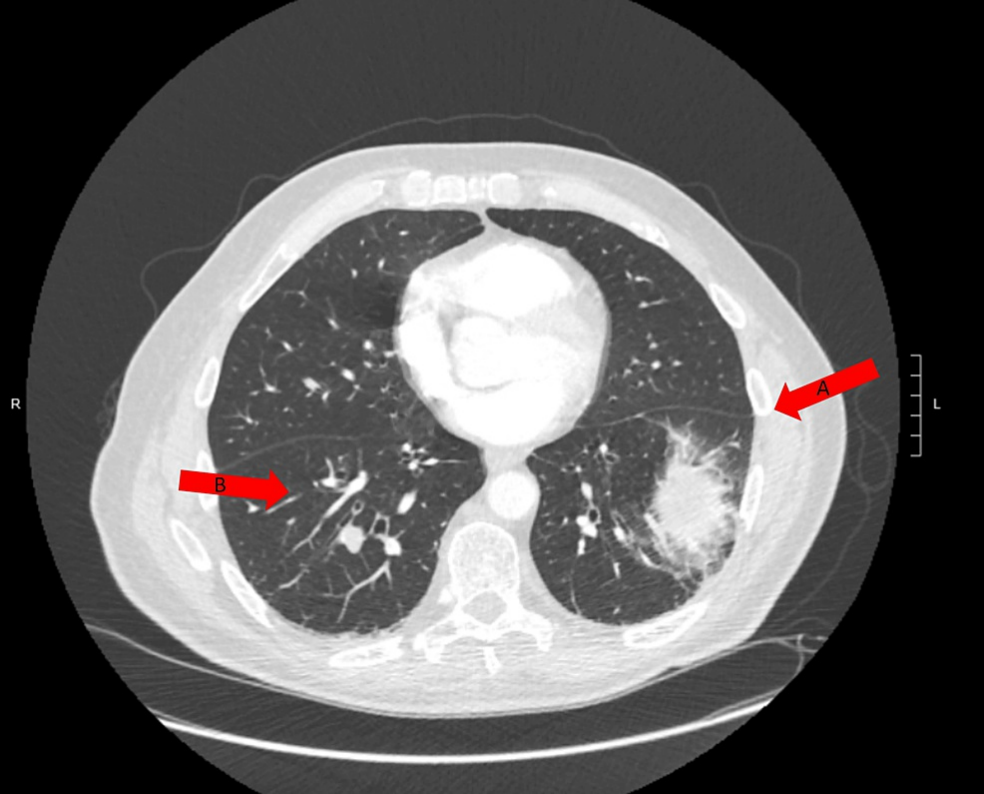
CT chest PE protocol showing left lower lobe pneumonia (arrow A), and multiple filling defects in the pulmonary arteries in the right lower lobe of the lung (arrow B) PE - pulmonary embolism

Upon initial assessment, his oxygen saturation was 89% on pulse oximeter, he was tachypneic with a respiratory rate of 23; however, the remaining vitals and physical examination were unremarkable. Arterial blood gas showed pH 7.47, PO_2_ 68, PCO_2_ 35, and oxygen saturation of 96% on the nasal cannula. CT-angiography with pulmonary embolism (PE) protocol of the chest revealed bilateral pulmonary emboli and left lower lobe pneumonia with otherwise normal lung parenchyma (Figure 1).

The patient was admitted to the hospital with acute hypoxic respiratory failure requiring oxygen therapy. Dapsone was discontinued on admission, and he received 3% Methylene blue. Treatment of COVID-19 pneumonia and acute pulmonary emboli was also initiated. The patient received steroids, and remdesivir and was started on a heparin drip during his hospital stay that was later on switched to abixaban.

His clinical status improved, and he eventually maintained good oxygen saturation on room air. The pre- and post-treatment methemoglobin levels were 12.4 and 3.6 consecutively. The patient was discharged home with oral anticoagulant for treatment of provoked PE and dapsone was substituted with mycophenolate as treatment of bullous pemphigoid.

## Discussion

Methemoglobin occurs due to the oxidation of ferrous iron in hemoglobin to its ferric form, impairing its oxygen-carrying capacity [3]. Methemoglobinemia is caused by the presence of the non-functional methemoglobin leading to tissue hypoxia. It is a relatively uncommon cause of low pulse oximeter readings. Acquired forms are the most common, caused by exposure to chemical substances that either directly or indirectly promote hemoglobin oxidation [4]. Acquired methemoglobinemia can be induced by agents like nitrates, nitrites, and aniline and drugs such as dapsone, with the most common cause being dapsone accounting for 42% of all cases given that the risk of methemoglobinemia increases with the long-term use of dapsone along with higher doses [3,5].

There are three classes of congenital methemoglobinemia (cytochrome-b5 reductase enzyme mutation or structural abnormalities), but more often it is acquired through secondary exposure to drugs, chemicals, or from pathologic conditions like sepsis and sickle cell crisis [6,7]. Examples of acquired causative agents are benzocaine, lidocaine, dapsone, aniline, and other nitrates or nitrites. Ash-Bernal et al.'s retrospective case series showed that dapsone was the most common etiology (42% of all cases) with a mean peak methemoglobin level of 7.6%, exceeding the methemoglobinemia cut-off of 3% as defined above [3].

The risk of developing methemoglobinemia is found in elderly patients who have medical comorbidities (like renal failure, anemia, or HIV) compared to healthy adults who rarely develop clinically significant methemoglobinemia while being on oxidizing agents [6]. A recent case study was published on a critically ill, elderly COVID-19 patient who developed idiopathic methemoglobinemia. This case study postulated that novel coronavirus proteins found in the virus have the capability of altering hemoglobin’s structure, interrupting red blood cells’ ability to carry oxygen, thus, making the individual more prone to methemoglobinemia [8]. However, little literature is found on methemoglobinemia occurrence in elderly individuals who have COVID-19-related issues concomitantly with methemoglobinemia-inducing agents.

Diagnosing methemoglobinemia is crucial for prompt management. In the presented case, the suspicion of methemoglobinemia arose due to the discrepancy between the patient's clinical symptoms and pulse oximeter readings. Pulse oximetry underestimates tissue hypoxia severity in the presence of methemoglobin, as it measures oxygenated hemoglobin (oxyhemoglobin). Co-oximetry, which accurately measures methemoglobin levels, can be used as a screening method in which a serial measure can guide therapy [9]. Shihana et al. have reported a bedside blood quantitative test to determine methemoglobin using a color chart with an 88% agreement rate, the detection of methemoglobin was higher in blood samples with a methemoglobin concentration greater than 20% reflected by noticeably darker coloration [10]. In addition, the Evelyn-Malloy test can also be used as a confirmatory test in suspected methemoglobinemia [1]. Patients with methemoglobinemia can present with cyanosis that is out of proportion to oxygen saturation given the unique effect of methemoglobin on the standard oxygenation reading on pulse oxi-meter, a chocolate brown blood color is pathognomonic for methemoglobinemia. Overall, a clinical diagnosis of methemoglobinemia can be confirmed by arterial blood gas paired with pulse oximetry and serum methemoglobin levels [2].

The management of methemoglobinemia involves the administration of methylene blue, a reducing agent that converts methemoglobin back to functional hemoglobin. In the presented case, treatment with 3% methylene blue effectively reduced methemoglobin levels, leading to an improvement in hypoxia. However, ascorbic acid can be used as an alternative to methylene blue if there are any contraindications such as G6PD deficiency, or if it is not available [11,12].

Regarding the management of bullous pemphigoid, primarily it has been treated with topical corticosteroids and systemic can be added if necessary [13]. However, several medications are commonly used such as dapsone, in order to reduce systemic corticosteroid side effects or in the presence of a contraindication preventing their use [14]. In this case, the discontinuation of the offending agent, i.e., dapsone and substitution with mycophenolate aimed to ensure appropriate therapy for the underlying dermatological condition and reduce the risk of acquired methemoglobinemia.

## Conclusions

We describe a 73-year-old male who was evaluated for acute hypoxic respiratory failure related to multiple concurrent etiologies. Extensive workup showed that the patient had acute PE, COVID-19 pneumonia, and methemoglobinemia. He was successfully treated with methylene-blue, anticoagulant, steroids and remdesivir.

Given the significant morbidity and even mortality with both medical issues independently, this case highlights the importance for physicians to maintain a high index of suspicion for oxygen level discrepancy in hypoxic patients and consider the possibility of acquired methemoglobinemia in addition to more common causes of acute respiratory failure. This result in timely intervention and better overall patient outcomes.
